# Role of the Global Fund in national HIV/AIDS response in Myanmar: a qualitative study

**DOI:** 10.1186/s41256-021-00212-4

**Published:** 2021-08-09

**Authors:** Zarni Htun, Yingxi Zhao, Hannah Gilbert, Chunling Lu

**Affiliations:** 1 Department of Global Health and Social Medicine, Harvard Medical School, 641 Huntington Ave, Boston, MA 02115 USA; 2 Nuffield Department of Medicine, Centre for Tropical Medicine and Global Health, University of Oxford, Oxford, UK; 3 Division of Global Health Equity, Brigham and Women’s Hospital, Boston, MA USA

**Keywords:** Global Fund, Myanmar, HIV/AIDS, Health system strengthening

## Abstract

**Background:**

The Global Fund has been a major funding source for HIV/AIDS programs in Myanmar. In this qualitative study, we aim to understand the impact of Global Fund on national HIV/AIDS response in Myanmar during the era of Millennium Development Goals (MDGs).

**Methods:**

We conducted individual in-depth interviews by recruiting key informants through purposive snowball sampling. The respondents were engaged in the national/subnational response to HIV/AIDS in Myanmar and worked for the United Nations agencies, non-governmental organizations (NGOs), and civil society. Interview questions were organized around the role of Global Fund in strengthening national response to HIV/AIDS in the six building blocks of the Myanmar’s health system. Transcripts from the key informants were synthesized into specific themes through a deductive approach.

**Results:**

We found that the Global Fund has provided substantial support to (1) finance the national HIV/AIDS response in Myanmar, and (2) strengthen leadership and governance at the central level through improving coordination and collaboration, including more stakeholders (e.g. civil society, NGOs) in decision making process, and catalyzing policy changes on scaling-up key interventions. Yet, its role remains limited in addressing new demands at the township level in terms of capacity building, staffing, and medical supply resulting from rapid scale-up of HIV interventions and decentralization of service delivery in the public sector.

**Conclusion:**

There was a missed opportunity for Myanmar to capitalize on the use of the Global Fund’s funding to strengthen the health system. Deliberate planning is required to optimize the use of those scarce resources to provide universal coverage for HIV/AIDS.

**Supplementary Information:**

The online version contains supplementary material available at 10.1186/s41256-021-00212-4.

## Background

The Global Fund to Fight AIDS, Tuberculosis and Malaria approves grants based on demand-driven, country-led proposals. Since its inception, the Global Fund has intended to support programs for targeted diseases “in ways that will contribute to strengthening health systems” [1], and health system interventions were usually added to or embedded into disease-specific proposals [2, 3]. Despite these intentions, the Global Fund’s investments in supporting health systems were sometimes constrained by its poor alignment and harmonization with the existing systems [4]. Previous studies showed that the initial stages of the Global Fund’s funding, especially in its early stages, often created parallel processes that led to overlapping and duplication with those of the existing systems [5–7], while with scaling up of implementation process, countries usually see more positive contribution from the Global Fund through support, alignment and harmonization with existing health systems [8].

### HIV in Myanmar and the Global Fund’s involvement

**Fig. 1:**
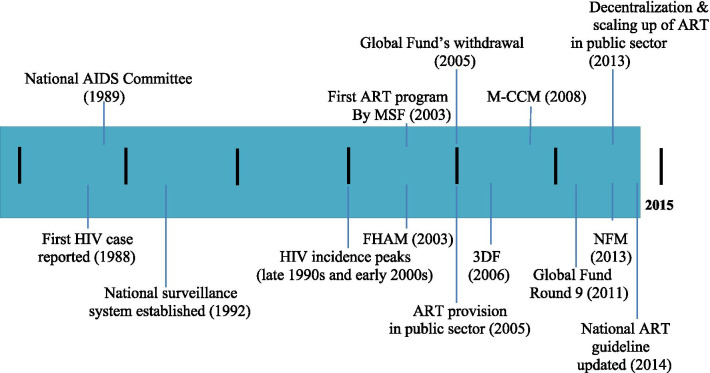
Historical timeline of the national response to HIV/AIDS in Myanmar (1985–2015). *Note*: 3DF, Three Diseases Fund; ART, Antiretroviral therapy; FHAM, Fund for HIV/AIDS in Myanmar; M-CCM, Myanmar country coordinating mechanism; MSF, Médecins Sans Frontières; NFM, New funding model

Myanmar is a country with a high burden of HIV/AIDS, tuberculosis (TB), and malaria [9]. Estimates from 2016 suggest that Myanmar had an HIV incidence rate of 450.3 per 100,000, which far exceeded the global average of 25.4 per 100,000 [10]. HIV/AIDS disproportionately affects vulnerable populations in the country. The Myanmar government formally launched the national response to HIV/AIDS in 1989 by establishing the National AIDS Committee, a high level multi-sectoral committee [9]. Its response to HIV/AIDS was undermined by a reluctance to acknowledge the epidemic in earlier years. In the late 1990s and early 2000s, an alarmingly high HIV/AIDS incidence was reported, and pressure from the international community gradually led to a shift in the military regime’s policy towards HIV/AIDS [11]. The change allowed for an expansion of the political space for the national response to HIV/AIDS and the influx of funding from international organizations [12]. In 2005, Myanmar received its first grant from the Global Fund for HIV/AIDS, TB, and malaria (Round 3, originally $54.3 million proposed for 2004–2008). However, the five-year grant was soon terminated in August 2005 due to restrictions imposed by the Myanmar government on international agencies, non-government organizations (NGOs) and civil society organization (CSOs) [13]. This included travel restrictions and the installation of lengthy and time-consuming procedures to obtain domestic travel permits [14] as well as formal or informal restrictions at the field level. In response to the Global Fund’s withdrawal, other international donors established a multi-donor funding mechanism named the Three Diseases Fund in 2006 (later re-named Access to Health Fund in 2018). Despite this effort, the limited scale of the funding was insufficient to meet the country’s needs [15]. The public sector—the main actor in the national response to HIV/AIDS—was only able to provide less than 10% of total antiretroviral therapy (ART) in 2005 [16]; and ART coverage remained one of the lowest in the world. In 2011, Myanmar started its re-engagement with the Global Fund and its application to the Global Fund (Round 9) was successful. Consequently, the Global Fund returned to Myanmar to support implementation of the National Strategic Plan on HIV/AIDS (NSP) (2011–2015) through Round 9 (2011–2015, $157.7 million requested) and New Funding Model (2013–2016, $210.8 million requested) See Fig. 1 for a historical timeline of the national response to HIV/AIDS in Myanmar.

**Fig. 2:**
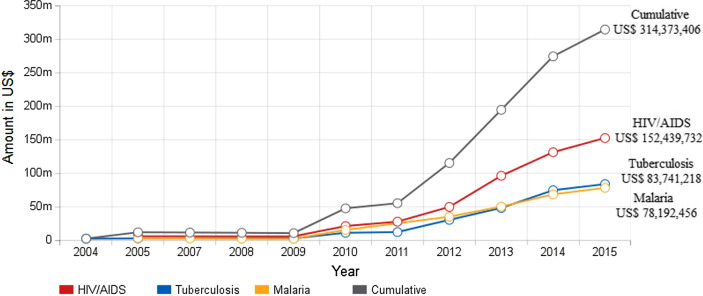
The Global Fund's disbursements to Myanmar. *Source*: The Global Fund. The Global Fund: Myanmar—Investments & results. Retrieved December 8, 2015, from http://www.theglobalfund.org/en/portfolio/country/results/?loc=MMR

Since 2011, the Global Fund has been the single largest financing source for the national HIV/AIDS response in Myanmar, contributing over $22 million (56% of the total spending) in 2012 and over $26.8 million (50% of the total spending) in 2013 (Fig. 2). The Global Fund has committed its financial contributions (US$ 160 million) to the national HIV/AIDS response for the period 2013–2016. This allowed Myanmar to emphasize the scaling-up and decentralization of service provision in public sector for ART, HIV counseling and testing, and harm reduction among injection drug users. External sanctions and the “zero cash policy” of the Global Fund, i.e. “the condition in which no national entities would receive any cash, but instead, the Principal Recipient would undertake all purchases and payments directly” restricted the direct fund flow to the Myanmar government [17]. Therefore, the fund flow was divided between the two Principal Recipients: Save the Children International which is the Global Fund’s Principal Recipient for programs run by NGOs, and the United Nations Office for Project Services (UNOPS), which is the Global Fund’s Principal Recipient for public sector. At the national level, implementation was overseen and coordinated by Myanmar Country Coordinating Mechanism (now organized as the Myanmar Health Sector Coordinating Committee).

This scale of investment on HIV/AIDS, along with the plan to scale up and decentralize HIV service provision in the public sector, placed new demands on the health system. To understand the challenges in using foreign aid, we conducted a qualitative study on the role of Global Fund during the era of Millennium Development Goals (MDGs) in the country’s response to HIV/AIDS from a health system perspective.

## Methods

### Study framework

We investigated the role of the Global Fund in Myanmar’s HIV/AIDS response from a health system perspective. In line with the World Health Organization (WHO), our definition of a health system is “all organizations, people and actions whose primary intent is to promote, restore or maintain health [18]. We used the WHO’s health systems framework which envisions health systems through six key building blocks that encompass leadership and governance, financing, medical products, vaccine, and technology, health information system, health workforce, and service delivery [18]. This framework has been previously used to analyze the interaction between global health initiatives and country health systems [19].

### Data collection

Individual, in-depth interviews were conducted in Myanmar from June to October 2015. Key stakeholders were purposefully selected, approached and invited to participate. Additional stakeholders were recommended or referred by the primary contacts. 15 interviews were conducted with key informants from UN agencies (4), international NGOs (9), and local NGOs/CSOs (2) and were engaged in the national HIV/AIDS response at the national/sub-national level. The diversity of the sample helps ensure both information richness, and inclusion of diverse ranges of perspectives on the health system—key hallmarks of purposeful sampling [20]. We did not include participants currently employed in the public sector, i.e. Ministry of Health and Sports, due to time and resource constraints. For example, it could take many months to obtain administrative approval to interview employees of the public sector. Data collected in this study, therefore, represented the experiences of those working in private and international organizations. Our interviewees had previous experience working across multiple sectors and freely shared insights from their prior experiences working within the public sector.

Using a semi-structured interview guide (Additional file 1), the study team asked respondents questions regarding their experiences about the role of the Global Fund in the national response for HIV/AIDS based on the six building blocks of health systems. Eight interviews were conducted in English and seven were conducted in the local Myanmar language. All interviews lasted 60–90 min in duration, were conducted in a private, quiet space of the interviewee’s choosing, and were audio-recorded with permission. All recordings were subsequently transcribed and translated into English.

### Data analysis

All personally identifiable information of the respondents (the name of the respondents and the names of their position and organization) was removed from the transcripts and replaced by unique anonymous codes. A deductive approach—testing theory and framework using the data [21] was applied to analyze the transcripts based on the six building blocks of the health system. Responses from the key informants were categorized into themes related to each building blocks. Within each of the pre-determined building block themes, sub-themes were identified by inductively examining the data using a thematic, conventional content analytic approach [22]. These more specific concepts further described each building block; each concept was refined through an iterative process and was supported with illustrative quotes. The final themes were constructed into a logical structure through an iterative process that reflected respondents’ experiences and opinions.

### Ethics approval

The study was approved by the Ethical Review Committee (ERC) of the Department of Medical Research of the Ministry of Health and Sports Myanmar, and Harvard Longwood Medical Area Institutional Review Board. Written informed consent was collected from all participants.

## Results

Key informants articulated that the Global Fund’s financing has allowed the national HIV/AIDS response “to grow, to expand, and to learn”, but stakeholders had to work on the process “with a lot of pain, headaches, [and] hiccups” (R04). Results were organized around the six building blocks—A–F, as described below and shown in Table 1.

**Table 1:** Summary of result findings by health system building blocks

Building block	Sub-themes and major concepts
Financing	Largest funding source for HIV/AIDS response Criticism of “zero cash flow policy” Reliance of donor funding and limited domestic financial resource
Leadership and governance	Alignment and coordination between donors, public sector, NGOs and civil society Trigger of important policy changes Engagement of NGOs and civil society in service delivery and national coordination Challenge in keeping all stakeholders involved and decision-making, especially at township level
Medical supply	Main supplier for related medicine Parallel procurement and supply chain system created inefficiency in medicine provision
Health information system	Improved data management and monitoring capacity of Ministry of Health and Sports Improved monitoring and evaluation capacity of implementing agencies Limited capacity at township level
Health workforce	Lack of staffing in public sector hindered scale-up of interventions Frequent turnover and lack of power for recruitment and deployment in hospitals Limited funding for workforce training
Service delivery	Prevention activities lacked focus on men who have sex with men and challenges in implementing harm reduction activities Care and support activities with challenges in stigma and hard-to-reach population Treatment scale-up: benefit and challenges of decentralization Fragmented service delivery system and inefficient coordination between public and non-public stakeholders

### Financing

All respondents acknowledged the crucial role that the Global Fund played in financing the national response for HIV/AIDS as the single largest funding source. However, some respondents voiced criticism of the Global Fund’s “zero cash flow policy”, which made the financing in “a bit of convoluted way”: “cash doesn’t come to the government programs” and “somebody else in the parallel process [is] managing finances for them” (R03). Some organizations utilize a system of disbursement called “the Managed Cash Flow” where they deploy a cadre of staff known as ‘field finance assistants’ to every state and region. Having cash in hand, field finance assistants make direct disbursement to service providers in the public sector through advance payment or offer cash reimbursement (based on the work-plan and implementation of activities). Some respondents highlighted system constraints at the operational level, especially when the field finance assistants were not able to check with service providers about the eligibility of expense reimbursement.

She [field finance assistant] reimbursed straight to the staff or sometimes into the hands of Township Medical Officer [TMO]. Reimbursement was a hundred percent… I am not sure what kind of vouchers they show to the finance staff… But she [finance staff] cannot argue with the TMO. If there is a problem, she can be, I mean, she can be sacked. That is kind of threatening. (R07)

Respondents also noted that the Global Fund’s zero cash flow policy did not help the MOHS improve their financial management capacity and accountability*.* While the government has increased its investment in HIV/AIDS in recent years, the level of the government’s financing remains low in terms of overall health expenditure. Respondents voiced concerns over Myanmar’s excessive reliance on Global Fund and other foreign donors, which posed a significant challenge to the financial sustainability of current activities. One respondent (R06) remarked that “the government does not have sufficient tax income to fund their own AIDS response.” This indicated the government’s inability to sustain the current momentum of the national HIV/AIDS response with its own financial resources.

It is not going to be sustainable in the near future; that’s for sure unless Myanmar suddenly becomes a huge oil nation or whatever. I don’t see that happening. (R06)

### Leadership and governance

According to the respondents, in addition to financing, the most notable effect that Global Fund funding has had on the national HIV/AIDS response is the strengthening of its leadership and governance, particularly in the four key areas highlighted below.

#### Alignment and coordination

Key informants explained that the Global Fund funding played an important role in improving alignment and coordination between donors, the public sector, NGOs and CSOs. They emphasized that the Global Fund’s HIV/AIDS financing was aligned with the NSP. Respondents explained that program reviews conducted by the executive working group of Myanmar Health Sector Coordinating Committee or one of its Technical and Strategy Groups ensured the alignment and harmonization between the programs funded by the Global Fund and the national priorities and plans.

The influx of funding from the Global Fund introduced coordination mechanisms into the National AIDS Programme and optimized coordination for planning, proposal preparation (including that for the Global Fund), and NSP preparation and reviews. Respondents expressed their positive experiences of improved planning and coordination in the MOHS:

We remind everyone in the room, not just the government; we have to remind ourselves—‘Wait! Remember last year when we submitted the concept note, we received this feedback. Let’s think about how we can incorporate these interventions to strengthen our program to address, you know, the technical guidance provided to us.’ I think it is a good check-and-balance. (R01)

Respondents perceived that the coordination mechanisms of National AIDS Programme greatly improved transparency and information sharing among the stakeholders over time. This process also helped stakeholders gain each other’s trust and develop a culture of mutual support and collaboration. One respondent (R10) from non-public agency described this change in the nature of collaboration between government hospitals and civil society:

Right now, when we are going to organize trainings in a hospital, the hospital may arrange for it. They arrange a room for the training. They invite us. They welcome us. They collaborate with us in organizing some events. They also tell us to contact them directly if necessary and to tell them directly if we have so and so issues. (R10)

#### Policy development: the Global Fund funding makes it possible

Respondents pointed out that the availability of financing from the Global Fund triggered several important policy changes regarding HIV/AIDS in the recent years. These changes were not instituted by the Global Fund directly, rather local actors devised and set priorities that were aligned, and the inflow of funding from the GF allowed for the institution of these changes. With “push and pull” from the Global Fund funding, the NSP has been updated and regularly monitored and evaluated. For example, the updated NSP allowed for broader participation of stakeholders, most notably the civil society, in governance of the national response. Recent updates (2014) of the NSP and the national guidelines for clinical management of HIV/AIDS have also signaled significant policy changes in terms of the country’s emphasis on treatment, especially scaling up ART, through standardization of essential service packages and simplification of the ART regimens. One respondent described the catalytic effect of the Global Fund’s financing in these terms:

[I]t can be the leverage—so a bit like judo, you know; use the weight to the other. They [the Global Fund] put their money in. Because of that, we needed to reform the guidelines on treatment in the country. (R03)

Key informants also remarked that the country has gained positive experiences of grant implementation in compliance with the Global Fund’s requirements and standards over time. Domestic stakeholders are willing and ready to accept technical guidance and inputs provided to them. Respondents remarked that the presence of the Global Fund’s financing triggered these policy developments, spurring changes in the public sector that are poised to substantially improve the welfare of people infected or affected by HIV/AIDS.

#### Engagement of stakeholders: NGOs and civil society

The Country Coordinating Mechanism of the Global Fund ensures involvement of NGOs and CSOs in proposal preparation and governance of the national response to HIV/AIDS. In addition to the scaling-up of interventions funded by the Global Fund, participants noted that local NGOs and CSOs have been playing an increasingly important role. In the past, the role of local NGOs was mainly limited to providing counseling and home-based care, but now they have become engaged in providing services such as testing and patient follow-up. Additionally, representatives from the local NGOs have become increasingly engaged and confident in discussing various issues at the Myanmar Health Sector Coordinating Committee. A key informant of a local NGO explains:

In the past, we took a seat and were just sitting and listening. We didn’t dare to talk in front of the Chair—the Minister… Right now, we have to talk when it is really necessary… So gradually our participation became meaningful. (R09)

#### Challenges in planning and coordination

Respondents discussed the challenges related to coordination, notably ensuring that all stakeholders were well-informed and engaged in discussions and the decision-making process, which was described as time consuming and occasionally unclear. Participants explained that this lack of coordination was frustrating, especially for non-governmental stakeholders:

There are plenty of challenges in terms of ensuring that everybody has to say and everybody is informed… You know this proverb: “If you want to go fast, go alone. If you want to go far, go together.” … So given that, we try to get as many stakeholders as possible involved; it takes a long time. (R06)

Many respondents observed that stakeholder coordination was particularly challenging at township level. A key informant (R05) commented that the public sector’s commitment seemed to diminish at the local township level. However, the same respondent (R05) also clarified that this diminished role “is not because people [TMOs] do not want to do the business, but [because] they need support.” TMOs, who bear the responsibility for the implementation of different vertical programs, are sometimes not fully aware of all plans. In such cases, non-public partners face constraints in coordinating their activities with the public sector at the township level.

### Medical supply

With vertical programs supported by the Global Fund funding, the UNOPS and Save the Children International (the two Principal Recipients), run their procurement and supply chain systems in parallel. Several respondents highlighted the overall need in the public sector for strengthening the medical supply chain and logistics system, along with building the capacity of staff. At the township level, one key informant commented that inventory management systems were paper-based, creating a challenge at the local level for ensuring a consistent supply of key medications. Another explained that funding from the Global Fund should be used to prioritize assisting township hospitals by creating a clear and coordinated system for managing supplies of key materials.

In some places medicines may be piled up in stocks, but in other places, medicines are in shortage. The government’s medical supply chain for the whole country is based on the central store. The central store delivers [medical supply] upon request. So in some cases, there is no order and no delivery. I think there are also some constraints in this part…The Global Fund is important because the Global Fund has been supporting the medicines provided by NAP until now. And the Global Fund [funding] is also supporting all patients in the NGO sector. So we can say that the Global Fund [funding] is supporting more than 100,000 patients currently receiving ART. (R12).

### Health information systems

According to the key informants, funding from the Global Fund has improved the availability and reliability of strategic information about the HIV/AIDS epidemic. Data management and monitoring capacity of the implementing agencies have improved because of the Global Fund grants’ emphasis on data quality and monitoring. This also reflects the willingness and ability of domestic agencies to adapt and comply with the Global Fund’s fundamental requirements over time. A respondent (R04) remarked:

Ten years ago you could not talk about data. Ten years ago, many people from outside or inside didn’t trust the data that we have or from MOHS. Now all partners trust the data we have because there is transparency of the way of working. (R04)

Key informants also acknowledged the Global Fund’s monitoring & evaluation mechanism in improving the capacity of implementing agencies in health information systems. One noted:

They [the Global Fund] are very precise about data. They check everything including the sources. As they are doing so, I would say that the skills of the volunteers, of our staff at different levels, and of staff from the health department have improved than before… They are always monitoring us and also teaching us for improving the quality. (R13)

At the township-level, however, monitoring and evaluation systems in the public sector remain paper-based and need to be improved. Respondents noted that application of modern technologies (e.g. electronic database, computers, and internet) is limited at the township level due to lack of human resources, equipment, and technical support.

### Health workforce

Respondents agreed that one of the most urgent problems facing the health system is the lack of adequate staffing and capacity building, most notably in the public sector at the sub-national level. An inadequate workforce hindered the scaling-up and expansion of key HIV/AIDS interventions. According to respondents, MOHS officials at the central level were often burdened with outsized responsibilities for undertaking parallel or multiple tasks. At the township hospitals, the shortage of health workforce was even more pronounced. Respondents explained that many township hospitals lack key personnel—including pharmacists to oversee medicine stocks, and specific monitoring & evaluation personnel to manage database operations. In most cases, these specialized tasks fall to doctors and nurses:

We have built hospitals. Equipment is provided. We have labs but there are no technicians. It is because the soft component [human resources] is totally deficient. (R10)

Key informants also highlighted other challenges in township hospitals, such as frequent turnover of medical doctors, especially in remote areas. Turnover is exacerbated by the fact that township hospitals do not have the authority to recruit and deploy medical staff, making it difficult to fill positions and ensure adequate staffing. These staffing challenges have hampered timely and effective rolling-out of key programs:

So, just after we have given them trainings, they move [to another place]. What happens is that we give them trainings, and then they move. (R14)

Respondents commented that training supported by the Global Fund funding enabled health care workers and volunteers to acquire some skills and capacity. Some respondents were critical of donors’ hesitancy (including the Global Fund) to invest in long-term human capacity development and concerned about programmatic sustainability.

To address these issues, some short-term arrangements have been made to meet the needs of township hospitals. For example, technical staff (such as technical officers, pharmacists, and logisticians) were hired through the WHO and some NGOs and supported the related services in the township hospitals. This approach provided temporary support for the ART provision at the township hospital but does not address the overall staffing issues in the public sector.

### Service delivery on HIV/AIDS

The Global Fund’s financing significantly improved HIV interventions, especially in scaling-up and decentralization of key interventions such as ART. However, respondents commented that the government failed to fully utilize the funding from the Global Fund to ensure coverage of prevention, care and support, and treatment for vulnerable populations due to several challenges, which are described below.

#### Prevention

As guided by the NSP, prevention has focused on key populations including people who inject drugs (PWID), sex workers and their clients, and men who have sex with men. Main preventive care includes harm reduction for PWID (e.g. distributing sterile needles and syringes to break the chain of HIV transmission among PWID), condom promotion, prevention of mother to child transmission (PMTCT), awareness and education, etc. In 2013, nearly US$ 11 million (20% of the total expenditure on HIV/AIDS) was spent on prevention and the Global Fund contributed US$ 3.8 million.

Respondents commented that most governmental prevention programs were tailored toward the general population (e.g. condom promotion, education campaign,) and lacked programs targeting men who have sex with men. Some respondents felt that—even for the general population—current programs on awareness and mobilization were inadequate, and the level of public awareness about HIV/AIDS remained low. One respondent explained that this situation exacerbated the existing burden of HIV/AIDS-related stigma in the community (R10).

Several key informants highlighted challenges in implementing harm reduction. One respondent noted that the idea of distributing needles and syringes “may be not traditionally accepted by the government” (R01). Current governmental programs prioritize methadone maintenance therapy over harm reduction interventions for drug users.

They are not showing their leadership on the issue… Some government officials want to work on methadone. Sorry! This doesn’t stop you sharing the needles… First line has to be needle-syringe program. (R03)

In some cases, local communities are reluctant to distribute needles and syringes, fearing it could promote injecting drug use in their area. Subsequently, several respondents suggested MOHS to “re-think” its approach towards prevention among drug users.

#### Care and support: promoting the patients’ welfare

Self-help groups and civil society networks are the main providers of peer supports at the community level. However, some volunteers faced constraints in conducting home-based follow-up because of stigma.

When we organized volunteers and told them to follow up our new patients, they said that the whole town had already known them [patients] as HIV-positive people.” (R13)

Respondents working for the networks also explained that it was challenging to reach patients in migrant and mobile populations:

Migrant and mobile populations do not stay in a township [for long]. Sometimes, the places they live are really away; they move far away from villages. They may live in woodlands. (R13)

#### Treatment: scaling-up provision of ART

Supported by funding from the Global Fund, the public sector adopted a two-pronged approach for ART scaling-up: increasing the number of main ART center countrywide and decentralizing some service provision to lower-level health facilities. The main ART centers are located primarily at specialist hospitals or hospitals at state/regional and district levels. These primary facilities are tasked with enrollment of new patients, initiation of treatment, and management of complex cases, whereas township hospitals provide follow-up services to stable patients for continuation of treatment. Respondents noted that the provision of ART treatment at township hospitals helped patients save time and mitigate the cost of accessing care.

When some patients arrived there [decentralized site], they realized it was near to their home. For those who used to get up at two in the morning, they might get up at six in the morning to go there… They got the same medication… So, some [patients] became satisfied. As they felt satisfied, the information was spread from one to another, and a few more patients showed up. (R14)

This process of decentralization in service delivery was viewed as both important and challenging. A number of respondents highlighted numerous barriers to the effective decentralization of treatment to township hospitals, including limited human resources, concerns about the quality of treatment in township settings, limited laboratory and medical supply chain and stock management capacity, and overall weak communication links with main ART centers, referral labs, and regional medical stores.

“We reach to the township level decentralized sites, in most places we have only one doctor in township hospitals such as township medical officer. These guys- they also have other activities under their management. There is no kind of additional support in terms of human resources, let’s say in terms of benefits, salary – no, nothing. It is kind of like adding another burden over their shoulders and no significant support is received. So, there are some decentralized sites that are functioning well because they receive supports from the partners. But in some places, there is no collaborating partner, and most of these places are not functioning.” (R05)

“There might not be actual decentralization, I mean…. For example, if it is an actual decentralization, there must be transfer of responsibility and decision-making authority to the lower level. But the lower levels do not make any actual decisions and they don’t have any decision-making power. We concern that it may still be controlled by the central level, as usual.” (R11)

The Global Fund’s implementing agencies faced security risks in trying to reach people with the greatest need, including the migrant population and those in remote and conflict-affected areas:

“Sometimes, INGOs [international non-governmental organizations] use illegal routes to get to the patients and could take a lot of risks to get there [conflict and border areas]. (R07)

In some cases, lack of information about availability of services widens the existing gaps of service utilization at the operational level. Due to these challenges, inequity in access to ART remains a significant challenge for the national HIV/AIDS response.

#### Other challenges

In addition to above issues, there were two more challenges in delivering prevention, care/support, and treatment: (1) fragmented service delivery system, and (2) inefficient coordinating between public sector and non-public stakeholders.

#### Fragmenting service delivery system due to vertical programs

The MOHS implements several vertical disease control programs (including HIV/AIDS funded by the Global Fund) and other public health programs through township health systems. These vertical programs run in parallel and speak to the fragmented nature of the health system. At the national level, the fragmentation has led to inefficiencies and weak coordination around cross-cutting issues:

You will become kind of like a ping-pong ball. So, the different national programs will play you around the circle, and at the end of the day, you get frustrated. (R05)

At the operational level, some respondents pointed out that implementing parallel projects constrained provision of integrated services. Patients could not obtain all needed services at a single delivery point, and therefore had to pursue care at multiple delivery points for different diseases. Health facilities in some areas are far from each other, which makes it very difficult for patients to obtain needed care. A respondent explained:

In terms of time spent by the clients, it is really challenging… So, because of that, we have a lot of … dropouts between the referral facilities. (R05)

#### Inefficient coordinating between public and non-public stakeholders

Participants acknowledged that NGOs and civil society have been playing substantial roles in the national HIV/AIDS response, especially in the places where the coverage of governmental health services is poor, such as conflict-affected areas. Coordinating the activities between the public and non-public stakeholders is challenging—yet vitally important for efficiently delivering services. Some key informants highlighted ‘ownership’ as a key problem at the township level. They explained that sometimes when NGOs intervened at township hospitals to provide temporary assistance and fill urgent gaps, this resulted in hospitals shifting responsibility to the NGOs. Respondents felt that the sense of ownership was vital to ensure sustainability of the programs in the public sector. Some key informants also pointed out the difficulties of collaboration at the local level.

We tried to run a one-stop shop in [Township X]… When we talked about it at the central level, it was going well. They agreed to it. But when we talked about it at the field level, we were not able to negotiate with the respective district medical officer [DMO]. (R12)

Respondents highlighted the important role that intensive advocacy from the MOHS could play in mitigating challenges at the local level by offering a “letter of collaboration.” A “letter of collaboration” refers to an official letter issued by the MOHS that instructs or informs local authorities to collaborate and support the non-public partners. One key informant said:

The process becomes smooth because of their support letter for collaboration. It is a little bit [more] convenient and easy to do prevention activities and find our targets in places like KTV [Karaoke Television] lounges and brothels in the township if we get their approval. (R09)

## Discussion

This qualitative study investigated the impact of the Global Fund financing on the national HIV/AIDS response from a health-system perspective. In the analysis of interviews with 15 key informants, we have two salient findings: (1) the rapid influx of HIV/AIDS funding from the Global Fund has allowed Myanmar to scale up HIV/AIDS response activities and resulted in a drastic expansion of ART provision and decentralization in the public sector. The national response to HIV/AIDS in Myanmar has been transitioning from an NGO-led to a government-led approach; the transition triggered a cascade of progress including improved leadership and governance at the central level (e.g. updated national guidelines) and strengthened technical capacity (e.g. establishing health information system). Scaling-up of ART created new demands on the fragile health system in Myanmar, and the role of funding from the Global Fund has been limited in addressing this challenge, particularly at the subnational level. According to the key informants, Myanmar has not fully leveraged the opportunity to use Global Fund financing to strengthen the national response on HIV/AIDS, especially to build capacity at the township level: the township health systems remain resource-deprived in terms of workforce, financing, and service delivery.

The findings in Myanmar are consistent with the opportunities and challenges associated with the Global Fund’s financing in other developing countries such as Bangladesh, Malawi, Mozambique, Zambia [2, 8, 23–26]: while rapid ART scale-up has a positive catalytic effect on a health system; it could also stress a fragile health system. Country stakeholders often raised concerns regarding donor dependency and financial sustainability of the programs beyond the global health initiative funding period [5, 6, 27–35]. Rapid scale-up of disease-specific interventions to achieve programmatic targets create parallel systems and processes, without adequate staffing levels, increase the workload and thus further burden the existing health workforce, which has already been overstretched in most cases [7, 29]. Some of these effects are most keenly exhibited at the sub-national level because this level has the fewest resources and their coordination structures are often undermined by the limited authority over decision-making and participation in planning and resource allocation processes. For instance, in Zambia and South Africa, the stakeholder coordination in implementation of HIV/AIDS programs was considerably lower at the sub-national level than at the national level in both countries [31]. Previous studies suggest that managing those challenges largely depends on whether or not a country has the leadership and management capacity to effectively coordinate the interactions between the foreign donors and domestic national and sub-national health systems [19, 36].

In Myanmar, according to our key informants, health financing from external sources is highly donor-driven, and in some cases the government is not in the position to set priorities or control over international aid. Bilateral/multi-lateral donors channel most of their funds through their own mechanisms in the country. While Global Fund proposals are led by CCMs, in Myanmar funds do not directly flow through the government system because of the “zero-cash policy”. Therefore, harmonizing activities between the Global Fund funding and the national HIV/AID response remain a challenge [37]. Key informants called for integrating the financial and technical capacity of the Global Fund into the existing health system so stakeholders could optimize the benefits of the Global Fund funding and strengthen the health care delivery platforms, especially at the township level. Meanwhile, donors should maintain continuity and predictability of their financing [38], realizing Myanmar’s current need for its national HIV/AIDS response.

The government of Myanmar has committed to attaining universal health coverage (UHC) by 2030 in its latest National Health Plan [39], which provides a golden opportunity for achieving the goals of the national HIV/AIDS response. Respondents believe that a rational step towards sustaining the national HIV/AIDS response is to ensure inclusion of HIV prevention, treatment, and care in the minimum package of services for UHC. This, however, would require major policy shifts, which must be supported by the government’s strong leadership and robust financing schemes. Currently, there is a significant gap between public investments and the funding necessary to sustain the national HIV/AIDS response. Key informants clearly expressed their concerns regarding the financial sustainability of the national HIV/AIDS response. In the long run, robust public financing is crucial for maintaining and expanding existing programs. Government spending on HIV/AIDS needs to be increased and must focus on building up delivery capacity at the township level. Myanmar should be inspired by the Abuja Declaration, in which African countries made a political commitment to allocate at least 15% of their annual national budgets to the health sector [40]. Public spending will play a paramount role in sustaining the national HIV/AIDS response, and strong political commitment is key to making this possible.

Despite being the first independent study of its kind to assess the impact of Global Fund on national HIV/AIDS response in Myanmar from a health system perspective, this research has the following limitations. First, the focus of the study was mainly on service provision and delivery at the central and local health systems, programmatic details on implementation was limited and the impact of Global Fund on population-level service coverage was not considered. The influence of contextual factors (e.g. political, economic, social, and cultural) on national HIV/AIDS response [25] was also not discussed. Future studies need to extend to these areas and further identify the barriers and facilitators for aid effectiveness. Second, current analysis did not include key informants from the public sector (Ministry of Health and Sports), or interview health care providers or patients due to time and resource constraints. The data in this study were collected from senior management and program officers in the private or international organizations. Third, data may be subject to potential bias due to purposeful sampling method or recall errors. We sought to minimize these types of bias by including a multivocal approach through the selection of informants from a range of institutions (UN agencies, and international/national NGOs and CSOs) and by asking respondents for references to particular events.

## Conclusion

This qualitative study investigated the impact of Global Fund funding on the national HIV/AIDS response in Myanmar. The study demonstrated that Myanmar has not fully utilized the opportunities offered by the Global Fund funding to strengthen its HIV service delivery system at the township level. Efficient use of funding and technical support from the Global Fund, other donors, and domestic resources will be key to unlocking these opportunities. The study contributed to the global depository of knowledge about the constraints and challenges in maximizing positive synergies between foreign donors and health system strengthening in developing countries.

## Supplementary Information

**Additional file 1**. Key informants interview guide

## Data Availability

The data underlying this article cannot be shared publicly for the privacy of individuals that participated in the study. The data will be shared on reasonable request to the corresponding author.

## Abbreviations

ART: antiretroviral therapy
CSO: civil society organization
MDG: Millennium Development Goal
MOHS: Ministry of Health and Sports
NGO: non-government organization
NSP: National Strategic Plan on HIV/AIDS
PWID: people who inject drugs
TMO: township medical officer
UNOPS: United Nations Office for Project Services
WHO: World Health Organization
