# Supplementary material for: Role of the Global Fund in national HIV/AIDS response in Myanmar: a qualitative study
Source: Glob Health Res Policy. 2021 Aug 9;6:27. doi: 10.1186/s41256-021-00212-4 (PMC8351103; doi:10.1186/s41256-021-00212-4)
Supplement: Supplementary file 1 — Additional file 1. Key informants interview guide [file 41256_2021_212_MOESM1_ESM.docx]

**Additional file 1.** Key informants interview guide

Introduction

1. Thank the respondent.

Thank you for agreeing to meet and for allocating time for the interview.

2. Explain the research objectives and process to the respondent.

Review the project information sheet.

3. Explain to the respondent about the informed consent process. Review the informed consent form. Obtain the informed consent from the respondent.

4. Explain to the respondent about the confidentiality of the responses.

Identifying information will be kept confidential and all the responses will remain anonymous.

5. Remind the respondent that he/she can refuse to respond to some specific questions (if uncomfortable) or he/she may decide to stop/withdraw from the interview at any time. Remind the respondent to ask any question at any time (for clarification).

6. Obtain the respondent’s consent for audio-recording of the interview.

7. Start the interview process.

Topic: How the national HIV/AIDS response can strengthen the health system in Myanmar

Let’s start by telling me anything that you think is important to understand about the current situation of the national HIV/AIDS response in Myanmar?

I would like to learn more about the Global Fund HIV program in Myanmar.

How would you describe the role of the Global Fund in delivery of HIV prevention, treatment and care services in Myanmar?

To the best of your knowledge, can you describe the role of the Global Fund HIV program in delivery of non-HIV services [other health care services than HIV] in Myanmar?

Could you explain any challenges/constrains you experienced, regarding the Global Fund HIV program in Myanmar? Why do you think it is challenging?

I am particularly interested in learning about the relationships between the national HIV/AIDS response and the different components of the health system, especially in terms of service delivery, health workforce, procurement and supply chain management, health information system, financing, and governance of the public health system in Myanmar. And I am going to ask you a few details about it.

Can you describe to me how you understand the relationships between the national HIV/AIDS response and these different components of the health system in Myanmar, based on your experience?

[Use the probes: “Can you give me an example of your experience?” “Can you describe for me what that would look like in practice?” “Can you give me an illustration of that so I can better understand that relationship?” etc.]

Depending on the response and the profile of the respondent, ask one or more of the following prompts:

*–* Any relationship between the national HIV/AIDS response and the health service delivery

*–* Any relationship between the national HIV/AIDS response and the health workforce

*–* Any comment about the relation between the national HIV/AIDS response and the medical procurement and supply chain system

*–* How about health information system? Can you describe any relation between the national HIV/AIDS response and the health information system?

*–* How about leadership and governance. Can you describe any relation between the national HIV/AIDS response and the leadership/governance of the health system?

Let’s talk about sustainability!

How do you think of the long-term sustainability of the national HIV/AIDS response in Myanmar?

What kind of opportunities, do you see? What kind of challenges, do you see?

In your opinion, what kinds of approaches may address sustainability of the national HIV/AIDS response?

How do you see the role of the Global Fund in this regard?

Finally, can you tell me any recommendations that you consider crucial in policy formulation about the national HIV/AIDS response in Myanmar?

Do you have any other thoughts that you would like to share me regarding the topic?

Is there any question that you have for me? Please.

Thanks a lot. It is indeed a very fruitful interview! Thanks you very much!

Remark: This is open ended semi-structured questionnaires. The investigator may probe the respondents with more follow-up questions, depending on their response.
